# ABCC3 Expressed by CD56^dim^ CD16^+^ NK Cells Predicts Response in Glioblastoma Patients Treated with Combined Chemotherapy and Dendritic Cell Immunotherapy

**DOI:** 10.3390/ijms20235886

**Published:** 2019-11-23

**Authors:** Serena Pellegatta, Natalia Di Ianni, Sara Pessina, Rosina Paterra, Elena Anghileri, Marica Eoli, Gaetano Finocchiaro

**Affiliations:** 1 Laboratory of Brain Tumor Immunotherapy, Fondazione IRCCS Istituto Neurologico Carlo Besta, 20133 Milan, Italy; diianninatalia@hotmail.it (N.D.I.); sa.pessina@gmail.com (S.P.); 2 Unit of Molecular Neuro-Oncology, Fondazione IRCCS Istituto Neurologico Carlo Besta, 20133 Milan, Italy; rosina.paterra@istituto-besta.it (R.P.); elena.anghileri@istituto-besta.it (E.A.); marica.eoli@istituto-besta.it (M.E.); gaetano.finocchiaro@gmail.com (G.F.)

**Keywords:** NK cells, ABCC3, resistance, chemotherapy, glioblastoma

## Abstract

Recently, we found that temozolomide (TMZ) can upregulate the expression of the multidrug-resistance protein ABCC3 in NK cells from both glioma-bearing mice and glioblastoma patients treated with dendritic cell immunotherapy combined with TMZ, allowing NK cells to escape apoptosis and favoring their role as antitumor effector cells. Here, we demonstrate that CD56^dim^ NK cells expressing CD16^+^ are predominant in patients surviving more than 12 months after surgery without disease progression. CD56^dim^ CD16^+^ NK cells co-expressed high levels of ABCC3 and IFN-γ. Notably, not only basal but also TMZ-induced ABCC3 expression was related to a strong, long-term NK cell response and a better prognosis of patients. The identification of the single nucleotide polymorphism (SNP) rs35467079 with the deletion of a cytosine (−897DelC) in the promoter region of the ABCC3 gene resulted associated with a better patient outcome. ABCC3 expression in patients carrying DelC compared to patients with reference haplotype was higher and modulated by TMZ. The transcription factor NRF2, involved in ABCC3 induction, was phosphorylated in CD56^dim^ CD16^+^ NK cells expressing ABCC3 under TMZ treatment. Thus, ABCC3 protein and the SNP −897DelC can play a predictive role in patients affected by GBM, and possibly other cancers, treated with dendritic cell immunotherapy combined with chemotherapy.

## 1. Introduction

Mechanisms of drug resistance are preferentially related to cancer cells and attributed to several factors, including biological and molecular heterogeneity, rapid proliferation, and infiltrative ability.

Chemoresistance is one of the most relevant causes of treatment failure and impairment of the prognosis of patients affected by glioblastoma (GBM) [1]. The current standard of care is maximal surgical resection, followed by concomitant administration of temozolomide (TMZ), an oral alkylating agent, along with fractionated radiotherapy, followed by six cycles of adjuvant TMZ [2,3].

The mechanisms contributing to the resistance to TMZ include the repair of DNA damage by enzyme O6-methylguanine-DNA-methyltransferase (MGMT) in cancer cells and/or an increased expression of ABC-type multidrug resistance (MDR) proteins.

The methylation of MGMT is an independent favorable prognostic factor of TMZ sensitivity: the median overall survival (OS) among patients with MGMT methylation was 18.2 months compared with patients without methylation surviving 12.2 months [4].

Another mechanism by which GBM cells can achieve drug resistance is the active extrusion of TMZ and other anticancer drugs through the cell membrane by multidrug resistance proteins [5]. A study performed in our institution investigated the expression of different multidrug resistance proteins (MDR) proteins on GBM specimens confirming the expression of P-glycoprotein-1 (Pgp-1) and multidrug resistance-associated protein (MRP)-1 proteins [6,7,8], but also finding the presence of MRP-3 (ABCC3) and MRP-5 (ABCC5) [9]. Interestingly, a significant correlation between high levels of MRP3 mRNA and poor survival of GBM patients was also detected [10]. A limited expression of MRP3 (also named ABCC3), as protein and mRNA, was observed in normal tissues [10]; the mRNA was absent in normal brain [9], however, its presence was described in rat astroglia and microglia [11]. ABC proteins are also express in immune cells. In particular ABCC1 and ABCC2 expression were described in CD4^+^, CD8^+^ T, CD19^+^ B, and CD56^+^ NK cells [12]. Recently, ABCC1 has been also implicated in lipid presentation and iNKT activation [13].

ABCC3 expression and efflux activity have been described in leukemia cells [14]. Notably, ABCC3 expression was found to be modulated by genetic polymorphisms causing a limited response to chemotherapy in patients affected by myeloid leukemia [15]. Our recent data indicated that ABCC3 is expressed and upregulated in NK cells from glioma-bearing mice treated with TMZ [16]. We also found that NK cells from GBM patients can express basal levels of ABCC3 and this expression was modulated by TMZ administration in combination with dendritic cell (DC) immunotherapy (DENDR1 clinical study) [17].

Our study describes cellular and molecular mechanisms induced by TMZ regulating and inducing ABCC3 drug-resistance in NK cells in GBM patients enrolled in the clinical study DENDR1 [17]. The identification of an ABCC3 polymorphism associated with increased ABCC3 expression and patient survival can help to explain differences in immune cell resistance and activation and, consequently, in clinical responses.

## 2. Results

### 2.1. CD56^dim^ CD16^+^ NK Cells with Cytolytic Ability Increase in DENDR1 PFS > 12 Patients

**Figure 1:**
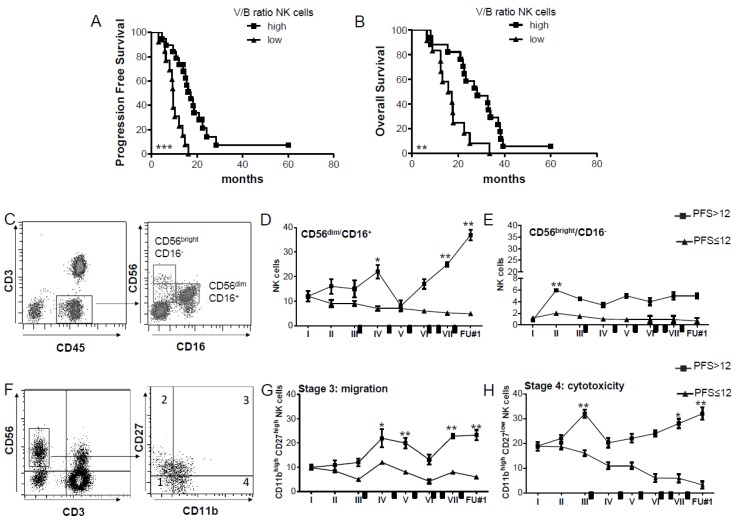
CD56^dim^ CD16^+^ NK cells with cytotoxic phenotype are prevalent in patients with PFS > 12. (**A**,**B**) Kaplan–Meier analysis curves of the correlation between V/B ratio of NK cell counts with (**A**) progression-free survival (PFS) and (**B**) overall survival (OS) (high V/B ratio > 2.1, *n* = 15 vs. low V/B ratio ≤ 2.1, *n* = 15). (**C**) Representative dot plots showing different subsets of NK cells based on the expression of CD56 as bright or dim and CD16. NK cells are gated in CD45^+^ CD3^−^ cells. (**D**,**E**) Time course of frequency of (**D**) CD56^dim^ and (**E**) CD56^bright^. NK cells measured by flow cytometry in PFS > 12 (*n* = 14) or PFS ≤ 12 patients (*n* = 16) (* *p* < 0.01, ** *p* < 0.005, vs. first vaccination, indicated as I). Data are presented as mean ± SEM. Black rectangles indicate temozolomide (TMZ) administration as maintenance. (**F**) Representative dot plot showing the four stages of NK cells by the flow cytometry evaluation of CD11b and CD27 expression. (**G**,**H**) Time course of frequency of NK cells from stage 3 or migratory stage (**G**), and stage 4 or cytotoxic stage (**H**) in PFS > 12 (black square, *n* = 14) or PFS ≤ 12 (black triangle, *n* = 16). (* *p* < 0.01, ** *p* < 0.005, vs. I vaccination). Data are presented as mean ± SEM. Black rectangles indicate TMZ administration as maintenance.

We previously described that the NK cell response was associated with a prolonged survival of GBM patients enrolled in the DENDR1 clinical trial, treated with dendritic cell (DC) immunotherapy combined with temozolomide (TMZ) [17]. We have extended the characterization to additional ten DENDR1 patients (Table 1), and the immunological evaluation confirmed that NK cell counts, after DC vaccinations, are strongly associated with prolonged survival (median progression-free survival (PFS) 17.2 months vs. 9.5 months, *p* = 0.0001; median overall survival (OS) 28.3 months vs. 16.4 months; *p* = 0.002, Figure 1A,B). We assessed the interferon (IFN)-γ expression by intracellular staining and flow cytometry on NK cells (Figure S1), observing a significant activation during the treatment in patients surviving more than 12 months without disease progression (PFS > 12) (Figure S1). A further characterization of the NK cell phenotype performed on 23 patients revealed that the CD56^dim^ NK cell subset expressing CD16, mainly responsible for the cytotoxic activity [18], is predominant in patients with PFS > 12 months (*n* = 10, Figure 1C,D). In particular, CD56^dim^ CD16^+^ NK cells displayed a positive modulation of their frequency when compared with basal level, exhibiting a significant expansion after the third, sixth and seventh vaccination that persisted after the treatment (Figure 1D). The frequency of the NK cell subset expressing CD56 but not CD16 (CD56^bright^ CD16^−^)—that are the primary source of the cytokine production [18,19] —increased after the second vaccination and remained relatively constant over the treatment (Figure 1E). The modulation of both the NK cell subsets was absent in PFS ≤ 12 patients (*n* = 13).

We also characterized the maturation subsets defined by CD11b and CD27 [20,21] previously tested in gated CD56^+^ CD3^−^ NK cells, during and after the treatment (Figure 1F–H). The separate evaluation of the four subsets revealed that stage 3 (CD27^high^ CD11b^high^) (Figure 1G), and stage 4 (CD11b^high^ CD27^low^) (Figure 1H), corresponding to NK cells with migratory and cytolytic activity, respectively, were positively modulated in PFS > 12 patients only.

### 2.2. The Multidrug Resistance ABCC3 Expressed by CD56^dim^ CD16^+^ NK Cells Is Active and Correlates with the Clinical Outcome

**Figure 2:**
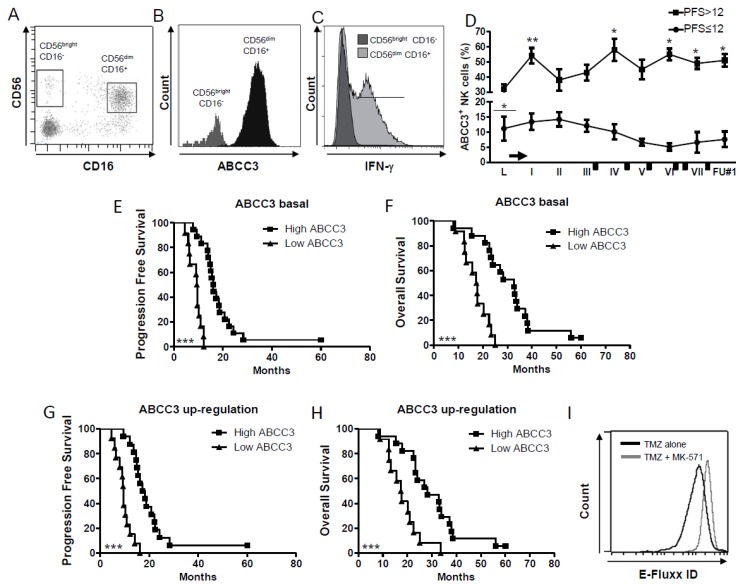
ABCC3 expressed by CD56^dim^ CD16^+^ NK cells is an indicator of better patient prognosis. (**A**–**C**). (**A**) Representative dot plots showing that CD56^dim^ CD16^+^ NK cells express (**B**) high levels of ABCC3, (**C**) and CD56^dim^ CD16^+^ ABCC3^+^ NK cells express IFN-γ. (**D**) Time course of frequency of NK cells expressing ABCC3 measured by flow cytometry (* *p* < 0.01, ** *p* < 0.005, vs. leukapheresis; underlined asterisk PFS > 12 vs. PFS ≤ 12). The arrow represents the standard Stupp protocol [3] after leukapheresis, before the first vaccination (indicated as I), and the black rectangles correspond to the TMZ administration as maintenance. (**E**,**F**) Kaplan–Meier survival curves showing the positive correlation between high basal ABCC3 expression in NK cells with (**E**) PFS and (**F**) OS. (**G**,**H**) Kaplan–Meier survival curves showing the positive correlation between ABCC3 upregulation during treatment with chemoimmunotherapy and better (**G**) PFS and (**H**) OS, (*** *p* < 0.001). (**I**). Flow cytometry displaying the multidrug resistance activity of NK cells treated in vitro with TMZ with or without the efflux inhibitor MK-571. Cells showing drug resistance have a MAF greater than 25%.

The multidrug resistance protein ABCC3 was described in our previous studies as a marker of NK cell resistance to TMZ in both the GL261 murine glioma model and DENDR1 patients [16,17]. The present, extended analysis of DENDR1 patients revealed that ABCC3 is strongly expressed by CD56^dim^ CD16^+^ NK cells compared to CD56^bright^ CD16^−^ NK cells (Figure 2A,B). Around 61% of the CD56^dim^ CD16^+^ ABCC3^+^ NK cell subpopulation expressed IFN-γ (Figure 1C).

The frequency of ABCC3^+^ NK cells assessed by flow cytometry at the time of leukapheresis confirmed a significant higher expression in PFS > 12 patients only (32.3 ± 3.0 vs. 11.2 ± 4.1 vs. PFS ≤ 12 *p* = 0.015; *n* = 10 and *n* = 13 respectively). A significant upregulation was assessed after radio-chemotherapy and DC vaccines and concomitant TMZ (Figure 2D). High basal expression of ABCC3 was associated with better PFS (median: 16.1 vs. 9.4; *p* < 0.0001, Figure 2E) and prolonged OS (32.8 vs. 17.5; *p* < 0.0001, Figure 2F). A significant correlation was also observed between ABCC3 upregulation during the treatment and prolonged survival (median PFS: 17.1 vs. 9.5; median OS: 28.3 vs. 17.5; *p* < 0.0001) (Figure 2G,H).

We also investigated the functional implications of ABCC3 expression in NK cells by testing their drug-resistant phenotype. CD56^dim^ CD16^+^ NK cells were enriched from PBLs of healthy donors and treated in vitro with 25 μM TMZ in the presence or absence of the selective MRP inhibitor MK-571. A flow cytometry assay based on a fluorescent substrate was used to investigate ABCC3 activity. An increased fluorescence intensity identified as a shift to the right of the flow cytometry histogram was indicative of the accumulation of the substrate as a consequence of the ABCC3 efflux block induced by MK-571 (Figure 2I). The multidrug resistance activity factor (MAF), an index of a resistant phenotype when higher than 25%, was 58.2% ± 7.6%. The fluorescence intensity decreased in the presence of DMSO, used as vehicle.

These data support the correlation between ABCC3 expression and NK cell resistance to TMZ.

### 2.3. The ABCC3 SNP rs35467079 Correlates with A Prolonged Survival

**Figure 3:**
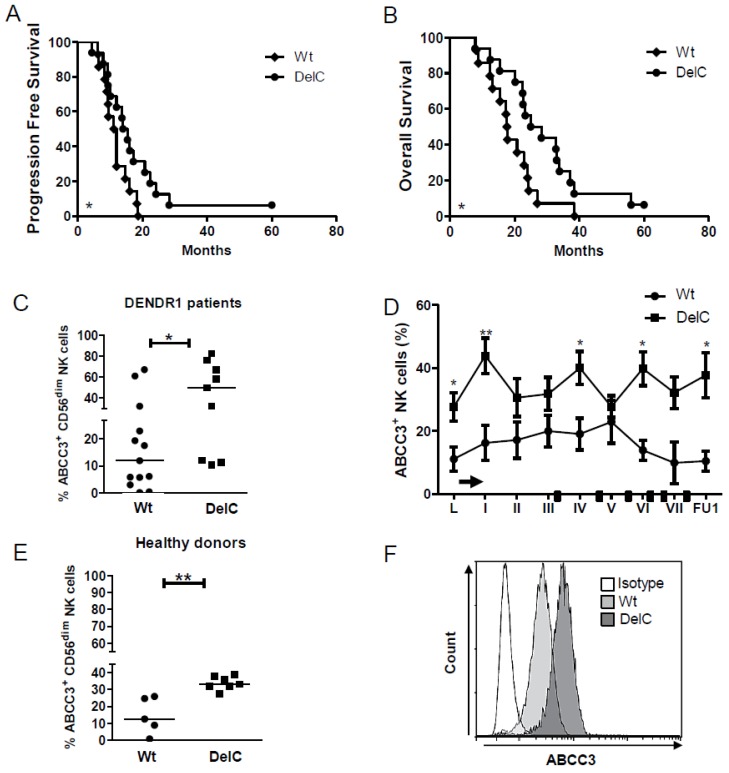
DelC genetic variant is related to a better response to chemoimmunotherapy. (**A**,**B**) Kaplan–Meier survival curves showing the correlation between DelC SNP and a good outcome, expressed as (**A**) PFS and (**B**) OS (DelC patients, *n* = 14, Wt patients, *n* = 16). (**C**) Scatter dot plots showing the frequency of NK cells expressing ABCC3 in a total of 23 patients (Wt or DelC), at the time of leukapheresis (L). (**D**) Time course of NK cells expressing ABCC3 measured by flow cytometry in DENDR1 patients carrying DelC compared to Wt. (**E**,**F**). (**E**) Scatter dot plots showing the frequency of NK cells expressing ABCC3 in 13 healthy donors divided in Wt (*n* = 6) and DelC (*n* = 7). (**F**) Representative histogram overlays for flow-cytometric analysis of ABCC3 expression on NK cells from healthy donors. The isotype control is represented as white histogram plot. The specific fluorescent signals are shown in light grey for Wt donors and dark grey for DelC donors.

Based on the observation that ABCC3 expression and modulation were higher in PFS > 12 only, we evaluated the presence of specific polymorphisms in the promoter regions influencing the expression and the activity of ABCC3 [22]. A total of 10 SNPs were investigated in the 5′-flanking region of the ABCC3 promoter [23], and 6 showed a minor allele frequency (MAF) > 0.05 in our patients. The SNP rs35467079 with the deletion of the −897 cytosine (DelC, −/−) was associated with a prolonged survival compared with the reference haplotype C/C, that we indicated as wild type (Wt) (median PFS: 14.7 vs. 10.8 months, *p* = 0.04; median OS: 26.6 vs. 17.7 months, *p* = 0.03) (Figure 3A,B). Notably, 81% of patients (13/16) with NK cell activation carried the rs35467079 SNP (*p* = 0.005 Fisher test) (Table S1).

We also observed that the basal frequency, at the time of the leukapheresis, of NK cells expressing ABCC3 assessed in 23 patients was significantly higher in DelC compared to Wt patients (45.8% ± 8.7%, *n* = 10 vs. 19.5% ± 6.1%, *n* = 13, respectively; *p* = 0.02) (Figure 3C). A positive modulation of the frequency of ABCC3^+^ NK cells was revealed during treatment in DelC patients only (Figure 3D). The count of NK cells expressing ABCC3 was significantly higher in DelC compared to Wt patients (920 ± 297 vs. 131 ± 67 count of positive cells/μL, respectively; *p* = 0.001). We also analyzed the expression of DelC in a group of healthy donors (*n* = 13), where 7 of them (58%) expressed DelC. In these donors, the expression of ABCC3 in NK cells was significantly higher than in Wt (33.7 ± 3.8% vs. 14.5 ± 10.7%, respectively; *p* = 0.001) (Figure 3E,F), a finding confirmed by evaluating the count of NK cells positive for ABCC3 (735.7 ± 372.0 in DelC vs.99.6 ± 88.2 count/μL of blood in Wt, *p* = 0.004).

These results support the potential role of this polymorphism in regulating ABCC3 expression in NK cells.

### 2.4. NRF2 Is Activated by TMZ Treatment in NK Cells Expressing ABCC3

We tried to predict specific transcription factor (TF) binding sites in the 5′ flanking regions of ABCC3 using different TF databases, such as Jaspar (http://jaspar.genereg.net/) and TRANSFAC (http://genexplain.com/transfac/). The DelC SNP has not reported as a TF binding site so far, but ENCODE (Encyclopedia of DNA Elements, https://genome.ucsc.edu/ENCODE/), shows that different chromatin modifications occur in the region surrounding DelC.

**Figure 4:**
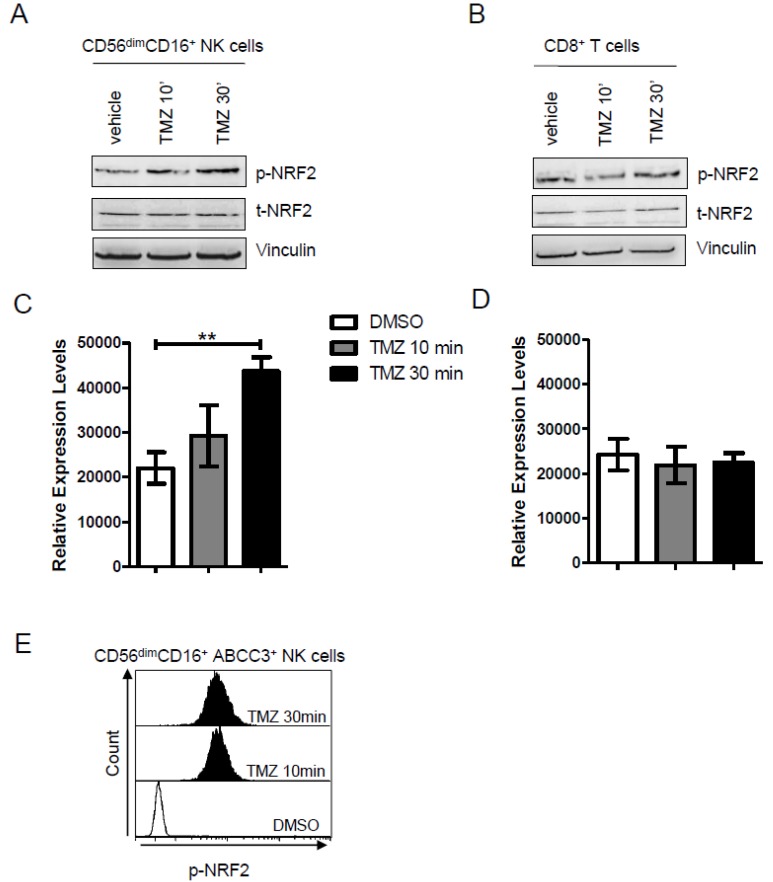
(**A**,**B**) Representative western blot analysis performed on enriched (**A**) NK and (**B**) CD8^+^ T cells from donor PBLs showing that 25 μM of TMZ increased the activation of NRF2 (phosphoSer40) after 30 min of treatment. Vinculin was used as loading control. The immunoblot is representative of three experiments. (**C**,**D**) Densitometric quantification of p-NRF2 expression in NK cells and CD8^+^ T cells treated with DMSO or TMZ at two different time points. Data are presented as mean ± SD of three independent experiments at the two different time points (*p* = 0.004). (**E**). Flow cytometry stacked histograms showing intracellular staining of p-NRF2 in donor-derived CD56^dim^ CD16^+^ ABCC3^+^. The DMSO treatment is represented as light grey histogram plot. The activation of NRF2 at 10 and 30 min is displayed in dark grey and black, respectively.

To define the signaling mechanism potentially involved in the ABCC3 regulation and induction in NK cells, we evaluated the transcription factor nuclear factor (erythroid-derived 2)-like 2 (Nrf2), as functional nrf2 response elements that have been described within the eighth intron of ABCC3 gene [24]. To accomplish this, we isolated PBLs from three healthy donors and enriched NK cells and CD8^+^ T cells that we used as negative controls. CD56^dim^ CD16^+^ NK cells and CD8^+^ T cells were treated with 25 μM TMZ or DMSO at different time points. Using immunoblotting, we observed a significant, time-dependent increase in pNRF2 expression in NK cells, but not CD8^+^ T cells, that were treated with TMZ, as compared with DMSO. The expression of total (t)-NRF2 protein did not change during the treatment. p-NRF2 expression was also investigated by the flow cytometry phospho-specific staining (Miltenyi Biotec) in NK cells enriched by donor PBLs treated with TMZ or DMSO. The NRF2 activation was confirmed in gated NK cells expressing ABCC3 both 10 and 30 min after addition of TMZ (Figure 4E).

The results provide mechanistic insights into the regulation of ABCC3 expression through NRF2.

## 3. Discussion

Novel, multiple mechanisms demonstrating the “innate” ability of NK cells to recognize and kill cancer cells, without antigen recognition, have been recently reported [25]. NK cells are “born to kill” and fight cancer, as recently reviewed by Wennerberg and Galluzzi [26], and occupy a key position in the complex network of interactions between innate and adaptive immune response. In our clinical data, we described that benefits from chemoimmunotherapy combination and gain of survival in patients affected by newly diagnosed GBM were essentially dependent on specific and long-lasting activation of NK cell response [17].

The present study represents a refinement toward a better evaluation of the complexity of NK cell antitumor response and an investigation of the molecular mechanisms of survival and drug resistance activation in NK cells after TMZ exposure.

First, we confirmed the cytotoxic features of NK cells by analyzing the CD56^dim^ and CD56^bright^ subsets. A significant difference was found in the CD56^dim^ frequency compared to CD56^bright^ NK cells from DENDR1 patients surviving without disease progression more than 12 months (PFS > 12), also defined as responders, in agreement with the criteria we had set in our clinical study (see study protocol in [17]). It is commonly accepted that the CD56^dim^ rather than the CD56^bright^ NK cell subset is responsible for the cytotoxic activity [18,27]. CD56^dim^ cells also display an early abundant IFN-γ production upon cytokine stimulation, in accordance with their effector ability [28].

Most CD56^dim^ NK cells in DENDR1 PFS > 12 patients expressed high levels of the low-affinity Fc receptor CD16, supporting their cytotoxic features [18,29]. A further characterization of the cytotoxic features of NK cells in DENDR1 PFS > 12 patients was performed by evaluating the four stages defined by CD11b and/or CD27 expression [16,20], and this revealed that the cytotoxic stage was predominant during the treatment and at the follow-up. The migratory stage is coherent with the massive tumor infiltration of NK cells in some DENDR1 patients who developed recurrence and underwent second surgery, as previously described [17].

The second important progress of this study was the observation that ABCC3 is expressed preferentially by the CD56^dim^ CD16^+^ NK cells, and these triple-positive NK cells also express high levels of IFN-γ. The ABCC3 transporter upregulated by NK cells during TMZ treatment is functionally active as demonstrated by an in vitro assay using the efflux inhibitor MK-571.

ABCC3/MRP3 is expressed in different normal tissues, including liver, intestine, skin [30,31,32]. When expressed by DCs, ABCC3 and the other MDR proteins are implicated in their migration at the inflammation site [33]. In lymphocytes, the activity of MDR proteins is related to cytotoxicity and their inhibition is implicated in the suppression of IFN-γ secretion [33,34]. Conflicting data are available about the active role of ABC transporters in extruding molecules such as TNF-α, IFN-γ, and perforins. It has been hypothesized that ABC-mediated transport of immune mediators across the plasma membrane results in autocrine/paracrine induction of intracellular signaling and consequent cell activation [35], however, ABCC3 expression and its involvement in chemoresistance in NK cells has not previously been reported.

The most relevant observation in our DENDR1 clinical study was that NK cells from PFS > 12 patients displayed higher basal expression of ABCC3, and its expression associated with a better prognosis. Based on the evidence that the expression and the activity of ABCC3 can be modulated by single nucleotide polymorphisms (SNPs), we have investigated and associated to the prognosis, the presence of six different SNPs [22], previously reported in normal cells, including liver or skin [23,32] and in leukemia [14].

The −897DelC, located in the 5′-flanking region, was the only SNP significantly associated with prolonged survival of DENDR1 patients. In addition, a significant positive modulation of NK cells expressing ABCC3 after TMZ administration, was observed only in DENDR1 patients with DelC (considering homozygous and heterozygous grouped together), supporting a potential role of this polymorphism in regulating ABCC3 expression in NK cells.

The increase of ABCC3 expression as a result of TMZ administration can also imply an induction of specific pathways and transcription factors. Previously, we found that during TMZ treatment, murine NK cells expressing Abcc3 do not undergo apoptosis and show a time-dependent activation of Akt [16], a key protein for immune cell survival. Since we were not able to confirm the same mechanism in NK cells from DENDR1 patients, we demonstrated the involvement of NRF2, a transcription factor (TF) already described as responsible for the ABCC3 induction under oxidative stress [24].

The main result from this study is the identification of patients who would derive a clinical advantage from chemoimmunotherapy and the characterization of the differences in immune cell resistance, their activation and, consequently, clinical responses. Benefits to other cancers can be investigated by considering the immunological aspect of specific chemotherapeutic agents. Imatinib mesylate (IM) is responsible for the increase and activation of NK cells when used to treat patients affected by gastrointestinal stromal tumors (GISTs) [36]. We can hypothesize a similar mechanism involving ABCC3 and inducing NK cell resistance, especially considering that the mechanism of resistance to IM also involves ABCC3 [37].

## 4. Materials and Methods

### 4.1. Patients and Treatment Protocol

Patients reported in this study, with first diagnosis of GBM and no IDH1-2 mutations, were enrolled in the two-stage Simon’s Design phase I–II clinical study DENDR1 (Clinical Trial of Immunotherapy with autologous tumor lysate-loaded dendritic cells in patients with newly diagnosed glioblastoma multiforme), EUDRACT n. 2008-005035-15. The study was approved by the Ethical Committee of Fondazione Istituto Neurologico Carlo Besta and from Istituto Superiore di Sanità (n. 18174(13)-PRE21-915, amendment 8 Nov 2013). Written informed consent was obtained from all participants.

Hypermethylation of the O6-methylguanine-DNA methyltransferase (MGMT) promote was evaluated by methylation-specific PCR as previously reported [38] (Table 1).

A total of 30 patients were considered: 20 patients were studied in the first stage [17], and 10 new patients enrolled in the second stage were considered for the evaluation of NK cell count and survival correlation and for the identification of the presence of SNPs starting from whole blood-derived genomic DNA. Flow cytometry analyses of NK cell subsets, ABCC3, and IFN-γ expression were performed on PBLs isolated by Ficoll density gradient centrifugation, before, during, and after the treatment when possible, available from 23 patients only.

All patients underwent leukapheresis and radiochemotherapy (RT/TMZ), according to the Stupp standard protocol [3]. Seven vaccinations were administrated as previously reported [17]. At each vaccine injection, clinical and immunological monitoring was performed. The first, fifth, sixth and seventh vaccinations contained 10 million DCs loaded with autologous tumor lysate; the second, third and fourth vaccinations 5 million DCs. Adjuvant TMZ was administered immediately after third vaccination and continued for six cycles.

### 4.2. Immunomonitoring

REAfinity^TM^ Recombinant Antibodies (Miltenyi Biotec, Bergisch Gladbach, Germany) were used for NK cell monitoring. Anti-CD56-FITC, CD3-PE-Vio770, CD45-VioBlue, CD16-PE were used to identify CD56dim and CD56 bright NK cells. The four stages were discriminated by using CD11b-APC-Vio770 and CD27-APC. ABCC3 expression was assessed before and after each vaccination as previously described [17], by using a primary antibody anti-ABCC3 (Thermo Fisher Scientific, Waltham, MA, USA) and a secondary anti-rabbit Alexa Fluor488 antibody (Abcam) according to manufacturer’s instructions. PBLs were then fixed and permeabilized using the Cytofix/Cytoperm solution (BD Biosciences, Franlin Lakes, NJ, USA) and intracellularly stained with an anti-IFN-γ (Miltenyi Biotec) antibody. NK cells were gated and then analyzed by flow cytometry for IFN-γ assessment. Acquisition of stained samples was performed using a MACSQuant (Miltenyi Biotec) flow cytometer, and data were analyzed using Flowlogic software (version 7.2, Miltenyi Biotec).

### 4.3. ABCC3 Transporter Activity

The transporter activity and the multidrug-resistant phenotype of ABCC3 were tested by the eFluxx-ID^®^ Green Multidrug-Resistance Assay (Enzo Life Sciences, Lörrach, Germany) in NK cells enriched starting from PBLs of healthy donors (CD56^+^ CD16^+^ NK Cell Isolation Kit, Miltenyi Biotec). Efflux activity of ABCC3 was assessed by flow cytometry with a fluorescent dye in presence or absence of the specific inhibitor MK-571. NK cells isolated from donor PBLs were treated with 25 μM TMZ or DMSO for 4 h in vitro. A multidrug-resistance activity factor value (MAF) was calculated as MAF = 100 × (F_MRP_ − F_CTRL_)/F_MRP_, where F is intensity of fluorescence. MAF values >25 are indicative of a positive multidrug resistance phenotype.

### 4.4. Western Blot

NK cell and CD8 T cell enrichment were performed using the CD56+ CD16+ NK and CD8+ T Cell Isolation Kit (Miltenyi Biotec), respectively. After magnetic cell separation, NK and CD8+ T cells were seeded in 6-plate wells cells at the density of 10^6^ cells/well and treated with 25 μM of TMZ or vehicle (DMSO), for 10 and 30 min, were washed with cold PBS and lysed in a buffer supplemented with protease and phosphatase inhibitors. Membranes with transferred proteins were incubated with the primary antibody anti-pNRF2 (phosphoSer40, 1:5000, Abcam), anti-NRF2 (1:1000, Abcam) or anti-vinculin (1:10,000). The primary antibody incubation was followed by incubation with peroxidase conjugated to the secondary antibody (anti-rabbit, 1:10,000). A chemiluminescence reaction using the ECL Plus kit (GE Healthcare, Chicago, IL, USA) was detected using G: BOX iChemi system (Syngene, Cambridge, UK).

### 4.5. DNA Extraction and Genotyping

Genomic DNA was isolated from blood samples with the use of Purogene Blood Core kit (Qiagen, Hilden, Germany) following manufacturer’s instructions. Ten polymorphisms located in regulatory region of ABCC3 gene were selected from the National Center for Biotechnology Information (NCBI) SNP database (https://www.ncbi.nlm.nih.gov/snp). The ABCC3 promoter was amplified by PCR using 100 ng of genomic DNA and FastStart Taq DNA Polymerase (Roche Basel, Switzerland) adding GC-rich solution to the mix. Four ABCC3-specific primer pairs (Eurofins Genomics, Ebersberg, Germany) were designed to avoid cross-recognition with homologous transporters. Purified amplicons were directly sequenced on an ABI 3130 sequencer (Applied Biosystems, Foster City, CA, USA) using the BigDye Terminator v1.1 Reaction Kit (Applied Biosystems) and analyzed with Chromas software. Oligo sequences for −897DelC FW: GAGAGCACTGACAAGCCCA; RV: CACATCACCTCGGCACGT.

### 4.6. Statistical Analyses

The ratio of the mean of second to seventh vaccinations/baseline values (V/B ratio) of NK cell count was calculated for each patient, and the median of all of the observations was used as the cutoff value to separate patients into the “low” or “high” groups.

The Wilcoxon signed rank test was used to test the significance of differences between markers at different time points. All *p* values were two-sided. The Fisher exact test was used to examine the differences in categorical variables among groups. The log rank test assessed differences in survival. All statistical analyses were performed using Prism 5.03 software.

## 5. Conclusions

Two are the main points of this study:

1. The CD56^dim^ CD16^+^ NK cell subset is responsible for a specific long-term antitumor immune response in GBM patients treated with chemoimmunotherapy. The positive modulation of these NK cells during and at the end of the treatment is associated with a better prognosis.

2. ABCC3 expressed by CD56^dim^ CD16^+^ NK cells play a relevant role in inducing resistance to TMZ and survival of NK cells. Increased ABCC3 expression is also correlated to a higher cytotoxic ability of NK cells, defined by IFN-γ expression, and is associated with prolonged survival of patients. The specific SNP DelC plays a positive role in ABCC3 expression and consequently in immunological and clinical response of the patients.

The demonstration of a predictive role of ABCC3 expression in NK cells, if confirmed on a larger number of patients, may have a relevant impact on selecting patients affected by GBM, and possibly other cancers, likely to obtain clinical benefit from chemoimmunotherapy.

## Figures and Tables

**Table 1:** Patient characteristics.

Patients (*n* = 30)	Age/Gender	MGMT (Met ≥ 0.1)	NK Cell Response ^§^	SNP	PFS (months)	OS (months)
1	55/F	U (0.07)	NO	DelC	13.7	22.5
2	62/F	U (0.01)	NO	Wt	12.0	24.4
3	66/M	U (0.04)	YES	DelC	15.4	15.4
4	70/F	U (0.00)	NO	Wt	14.7	17.8
5	49/M	U (0.00)	NO	DelC	10.2	12.5
6	65/F	M (0.71)	YES	DelC	20.8	33.9
7	60/M	U (0.01)	YES	DelC	9.3	25.0
8	58/M	U (0.00)	YES	DelC	9.4	22.6
9	50/M	U (0.00)	YES	DelC	16.1	33.0
10	48/M	M (2.38)	YES	DelC	4.4	7.8
11	23/F	U (0.003)	NO	Wt	3.1	6.4
12	44/M	U (0.03)	YES	DelC	24.2	38.4
14	62/M	M (0.46)	NO	DelC	7.9	20.2
16	70/M	M (1.50)	YES	DelC	17.2	32.8
19	56/M	U (0.00)	NO	Wt	3.2	6.9
20	48/M	U (0.00)	NO	Wt	9.0	12.4
21	53/F	M (0.47)	YES	DelC	28.3	56.0
22	63/M	U (0.02)	YES	Wt	6.5	8.1
23	45/M	M (0.74)	YES	DelC	>60.0	>60.0
24	55/F	U (0.00)	YES	DelC	14.0	28.3
25	M/58	M (0.18)	NO	DelC	22.4	37.2
26	F/45	U (0.07)	NO	Wt	12.0	17.6
27	M/49	M (0.38)	NO	Wt	9.5	13.2
28	F/54	U (0.00)	NO	Wt	12.0	15.5
29	M/43	M (0.56)	NO	Wt	9.4	17.3
30	M/65	M (0.26)	NO	Wt	18.7	27.0
31	F/60	M (1.78)	YES	Wt	10.7	22.9
32	M/62	M (0.21)	NO	Wt	16.1	33.5
33	M/53	U (0.04)	YES	Wt	18.3	38.5
34	M/49	M (2.39)	YES	DelC	12.0	23.3

Abbreviations: MGMT: O6-Methylguanine-DNA Methyltransferase, M: methylated; U: unmethylated; SNP: single nucleotide polymorphism; DelC: deletion of a cytosine; Wt: wild-type; PFS: progression free survival; OS: overall survival. § Significant activation of NK cell response evaluated as V/B ratio > 2.1.

## Supplementary Materials

Supplementary materials can be found at https://www.mdpi.com/1422-0067/20/23/5886/s1.
