# Supplementary material for: ABCC3 Expressed by CD56dim CD16+ NK Cells Predicts Response in Glioblastoma Patients Treated with Combined Chemotherapy and Dendritic Cell Immunotherapy
Source: Int J Mol Sci. 2019 Nov 23;20(23):5886. doi: 10.3390/ijms20235886 (PMC6928625; doi:10.3390/ijms20235886)
Supplement: Supplementary file 1 [file ijms-20-05886-s001.pdf]

Figure S1. NK cells activation lasts over time (after the end of the treatment in patients PFS > 12 only

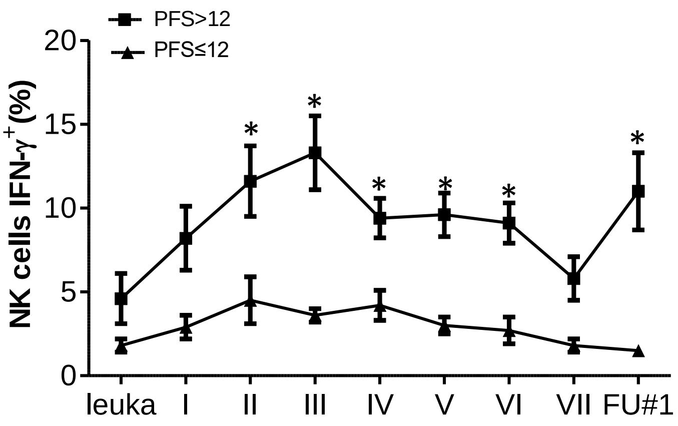

Time course of NK cells expressing IFN-γ evaluated by intracellular staining and flow cytometry. Patients were divided in surviving more than 12 months without disease progression (PFS > 12, n=14), and PFS ≤ 12 (n=16). A significant expansion of NK cells expressing IFN-γ was evident in patients PFS>12 only, already after the first vaccination (indicated as I in the graph), persisting during the treatment and at the follow-up (FU) (* $p < 0.01$ vs. leukapheresis [leuka]).

Table S1. Frequency of rs35467079 SNP in GBM patients

NK cell Response	Number of patients	Frequency (%)	
		Wt	DelC
NO	14	71.4 (n=10)	28.6 (n=4)
YES	16	18.8 (n=3)	81.2 (n=13)
